# Cellular localization of guanylin and uroguanylin mRNAs in human and rat duodenal and colonic mucosa

**DOI:** 10.1007/s00441-016-2393-y

**Published:** 2016-04-05

**Authors:** Øystein Brenna, Marianne W. Furnes, Bjørn Munkvold, Mark Kidd, Arne K. Sandvik, Björn I. Gustafsson

**Affiliations:** Department of Gastroenterology and Hepatology, St. Olavs Hospital, Trondheim University Hospital, Trondheim, Norway; Department of Cancer Research and Molecular Medicine, Norwegian University of Science and Technology, Trondheim, Norway; Centre of Molecular Inflammation Research, Norwegian University of Science and Technology, Trondheim, Norway

**Keywords:** Chromogranin A, Enteroendocrine cells, Guanylin, Tuft cells, Uroguanylin

## Abstract

Guanylin (*GUCA2A*/*Guca2a*/GN) and uroguanylin (*GUCA2B*/*Guca2b*/UGN) are expressed in the gastrointestinal tract and have been implicated in ion and fluid homeostasis, satiety, abdominal pain, growth and intestinal barrier integrity. Their cellular sources are debated and include goblet cells, entero-/colonocytes, enteroendocrine (EE) cells and tuft cells. We therefore investigated the cellular sources of GN and UGN mRNAs in human and rat duodenal and colonic epithelium with in situ hybridization (ISH) to determine co-expression with Chromogranin A (*CHGA*/*Chga*/CgA; enterochromaffin [EC] cells), defensin alpha 6 (*DEFA6*/*Defa6*; Paneth cells), mucin 2 (*MUC2*/*Muc2*; goblet cells) and selected tuft cell markers. *GUCA2A*/*Guca2a* expression was localized to goblet cells and colonocytes in human and rat colon. In human duodenum, *GUCA2A* was expressed in Paneth cells and was scarce in villous epithelial cells. In rat duodenum, *Guca2a* was only localized to goblet cells. *Guca2b* was focally expressed in rat colon. In human and rat duodenum and in human colon, *GUCA2B*/*Guca2b* was expressed in dispersed solitary epithelial cells, some with a tuft cell-like appearance. Neither *GUCA2A* nor *GUCA2B* were co-expressed with *CHGA* in human duodenal cells. Consequently, EC cells are probably not the major source of human GN or UGN but other EE cells as a source of GN or UGN are not entirely excluded. No convincing overlap with tuft cell markers was found. For the first time, we demonstrate the cellular expression of *GUCA2B* in human duodenum. The specific cellular distribution of both GN and UGN differs between duodenum and colon and between human and rat intestines.

**Electronic supplementary material:**

The online version of this article (doi:10.1007/s00441-016-2393-y) contains supplementary material, which is available to authorized users.

## Introduction

Guanylin (GN [gene name: *GUCA2A*]) and Uroguanylin (UGN [*GUCA2B*]) are endogenous ligands for the guanylate cyclase-C (GC-C [*GUCY2C*]) receptor. Heat-stable enterotoxin (ST) derived from *Escherichia coli* was first reported to elicit chloride and water secretion through guanylate cyclase and cyclic guanosine-3′,5′-monophosphate (cGMP) activation (Field et al. 1978; Hughes et al. 1978). The specific GC-C receptor for ST was cloned in 1990 (Schulz et al. 1990). *GUCY2C* (GC-C) is expressed throughout the intestinal epithelium from duodenum to colon in human, rat and other mammals (Krause et al. 1994; Qian et al. 2000). The endogenous ligands for GC-C were discovered in 1992 and 1993 (Currie et al. 1992; Hamra et al. 1993). Both *GUCA2A*/*Guca2a* (GN) and *GUCA2B*/*Guca2b* (UGN) are highly expressed in epithelial cells of the gastrointestinal (GI) tract (Beltowski 2001) and are secreted into the GI lumen but are also found in the systemic circulation (Date et al. 1996; Hess et al. 1995). GN and UGN are complementary in the GI tract, as GN increases in the cranio-caudal direction and vice-versa for UGN, possibly reflecting the pH optima for GN and UGN and also the resistance of UGN to chymotrypsin (Sindic and Schlatter 2006; Whitaker et al. 1997). Binding to GC-C in the intestinal epithelium leads to an intracellular increase of cGMP with subsequent activation of the cystic fibrosis transmembrane conductance regulator (CFTR), secretion of chloride and bicarbonate and inhibition of the sodium-hydrogen exchanger (NHE3), with resulting net water secretion (Field 2003). GC-C signaling has also been implicated in the regulation of satiety (Valentino et al. 2011), irritable bowel syndrome (IBS) and abdominal pain (Castro et al. 2013; Chey et al. 2012), tumor growth (Lin et al. 2010; Wilson et al. 2014) and the maintenance of intestinal barrier function (Han et al. 2011). Furthermore, a gain of function mutation has recently been discovered in a Norwegian family with chronic diarrhea and susceptibility to terminal ileitis (Fiskerstrand et al. 2012). GC-C agonists are now used in the treatment of constipation-dominated IBS.

Recently, we reported that *GUCA2A*, *GUCA2B* and *GUCY2C*, plus several steps of the GC-C signaling pathway, are down-regulated in inflammatory bowel disease (IBD). This may sustain a disrupted epithelial barrier, influence growth and epithelial renewal and possibly have implications in IBD pathogenesis (Brenna et al. 2015).

Since their discovery, the cellular sources of both GN and UGN have been investigated and debated. Both peptides have been linked to enteroendocrine (EE) cells in the GI tract. GN has been found not only in serotonin-producing enterochromaffin (EC) cells (Cetin et al. 1994; Hill et al. 1995) and in somatostatin-producing (D) cells (Ieda et al. 1998) but also in goblet cells and/or colonocytes (Cohen et al. 1995, 1998; Date et al. 1996; Lewis et al. 1993; Li and Goy 1993; Li et al. 1995) and in Paneth cells of the small intestine (Cohen et al. 1998; Date et al. 1996; de Sauvage et al. 1992). UGN has likewise been linked to EE/EC cells (Cui et al. 2000; Hess et al. 1995; Perkins et al. 1997), EC-like (ECL) cells (Date et al. 1999; Nakazato et al. 1998) and tuft cells (Kokrashvili et al. 2009).

As considerable divergence is present in previous findings, we aimed to investigate the cellular expression/localization of endogenous *GUCA2A*/*Guca2a* (GN) and *GUCA2B*/*Guca2b* (UGN) by means of a highly sensitive and specific in situ hybridization (ISH) method (Sordal et al. 2013) in human and rat duodenum and colon.

## Materials and methods

### Tissue

Human duodenal endoscopic pinch biopsies from healthy individuals and colonic endoscopic pinch biopsies from healthy controls included in a large IBD study, as approved by the Regional Medical Research Ethics Committee (approval no. 5.2007.910) and registered in the Clinical Trials Protocol Registration System (identifier NCT00516776), were collected. Whole duodenal and colonic sections from female Sprague Dawley rats (Taconic Farms, Hallingore, Denmark) weighing 200 – 250 g were extirpated under general anesthesia of the animals, which were thereafter killed by exsanguination. The general care and use of the animals were in accordance with the European Convention for the Protection of Vertebrate Animals used for Experimental and other Scientific purposes.

### In situ hybridization

Human and rat biopsies were fixed in 4 % formaldehyde for 3–5 days before being embedded in paraffin. Sections (4 μm thick) were mounted on slides and warmed for 1 h at 60 °C to ensure proper adhesion. Slides were deparaffinized in Neo-Clear, dehydrated in 100 % ethanol and air-dried.

ISH was performed by using the RNAscope 2.0 and 2-plex chromogenic assay kits (Advanced Cell Diagnostics, Hayward, Calif., USA) following the protocol provided by the manufacturer. All human and rat probes for ISH were designed and purchased from Advanced Cell Diagnostics. The probes used are shown in Table 1 with gene and corresponding peptide/protein names.

**Table 1:** Overview of in situ hybridization (ISH) probes used (gene names of ISH probes together with their respective peptide/protein name and accession numbers)

Gene name	Peptide/protein	Accession number
Human probes		
*GUCA2A*	Guanylin (GN)	NM_033553
*GUCA2B*	Uroguanylin (UGN)	NM_007102.2
*CHGA*	Chromogranin A (CgA)	NM_001275.3
*DEFA6*	Defensin, alpha 6	NM_001926.3
*MUC2*	Mucin 2	NM_002457.2
*DCLK1*	Doublecortin-like kinase 1	NM_001195415.1
*GNAT3*	α-Gustducin	NM_001102386.1
*SOX9*	Sex-determining region Y-box 9	NM_000346.3
*TRPM5*	Transient receptor potential cation channel	NM_014555.3
*INSM1*	Insulinoma-associated 1	NM_002196.2
Rat probes		
*Guca2a*	Guanylin (GN)	NM_013118.1
*Guca2b*	Uroguanylin (UGN)	NM_022284.2
*Chga*	Chromogranin A (CgA)	NM_021655.2
*Defa6*	Defensin, alpha 6	NM_001033076.1
*Muc2*	Mucin 2	XM_003749023.1
*Dclk1*	Doublecortin-like kinase 1	NM_053343.3
*Gnat3*	α-Gustducin	NM_173139.1
*Sox9*	Sex-determining region Y-box 9	XM_003750950.1
*Trpm5*	Transient receptor potential cation channel	NM_001191896.1
*Insm1*	Insulinoma-associated 1	XM_006224680.1

Serial sections were used for single-plex ISH. Adjoining sides of the sections were mounted facing up on the slides. After dehydration and air-drying treatment, the following steps were conducted: peroxidase blocker (*Pretreat 1*), gentle boiling (*Pretreat 2*) for 15 min and protease (*Pretreat 3*) for 30 min at 40 °C. After the pretreatment steps, the target probe was applied and hybridized for 2 h at 40 °C. Subsequently, the amplification steps (*Amp 1 – 6*) including application of a horseradish peroxidase (HRP)-linked labeling probe were performed prior to DAB (diaminobenzidine) visualization (10 min; DAB was provided by the manufacturer) and counterstaining with hematoxylin for 2 min. For duplex ISH, the slides were dehydrated in 100 % ethanol after *Pretreat 2* and air-dried again. Thereafter, Pretreat 3 was applied for 30 min at 40 °C. The target probe was subsequently applied for 2 h at 40 °C. As in single-plex ISH, signal amplification was performed in the next step. The 2-plex signal amplification system differs slightly from single-plex ISH. Amplification step 4 consists of a mixture of *Amp 4A and 4B* designed specifically for Channel C1 and Channel C2 target probes, which results in a different color reaction after application of the chromogens. Channel C1 uses HRP-linked (green/blue) and Channel C2 uses alkaline phosphatase (AP)-linked (red) labeling probes. The duplex ISH slides were counterstained in hematoxylin for 30 s, immersed in 0.02 % aqueous ammonia solution and air-dried at 60 °C for 15 min, before the mounting step.

### Immunohistochemistry

We performed immunohistochemistry (IHC) for GN (human) and Dclk1 (human and rat). Because of the lack of appropriate antibodies, we were not able to carry out IHC for GN (rat) or UGN (human and rat). Serial sections with adjoining sides facing up on the slides were used to determine the possible co-localization of *GUCA2B*/*Guca2b* (ISH) and Dclk1 (IHC).

GN IHC on human duodenal and colonic biopsies was performed with anti-GN (HPA018215; Atlas Antibodies, Stockholm, Sweden; dilution 1:50 or 1:100), as previously described (Brenna et al. 2015). Dclk1 IHC (ab31704; Abcam, Cambridge, UK; dilution 1:100) was similarly carried out, apart from antigen retrieval in citrate buffer, pH 6. Antibody detection was performed with secondary antibody (Dako REAL EnVision/HRP, Rabbit Mouse; Dako Denmark, Glostrup, Denmark). DAB (Dako REAL Substrate Buffer and Dako REAL DAB+ Chromogen; Dako Denmark) was used as chromogen. Hematoxylin was used for counterstaining.

### Cell counting

Whole sections from human duodenum were examined and the total number of GN (IHC)- or *GUCA2A* (ISH)-positive cells was separately counted in crypt and villous epithelium. For human and rat duodenum, the number of *GUCA2B*/*Guca2b* and *CHGA*/*Chga* expressing cells was counted in 4–8 non-overlapping visual fields (200×) of two representative sections.

### Image capture and processing

Images were captured by using a Nikon Eclipse E400 microscope (Nikon Instruments Europe, Amsterdam, Netherlands) and Nikon NIS-Elements BR 3.00 Imaging Software (Nikon Instruments Europe BV). Automatic white balance was used and the images were stored as TIFF-files. Figures were created in Adobe Photoshop CS5 (Adobe Systems, San Jose, Calif., USA).

## Results

**Fig. 1:**
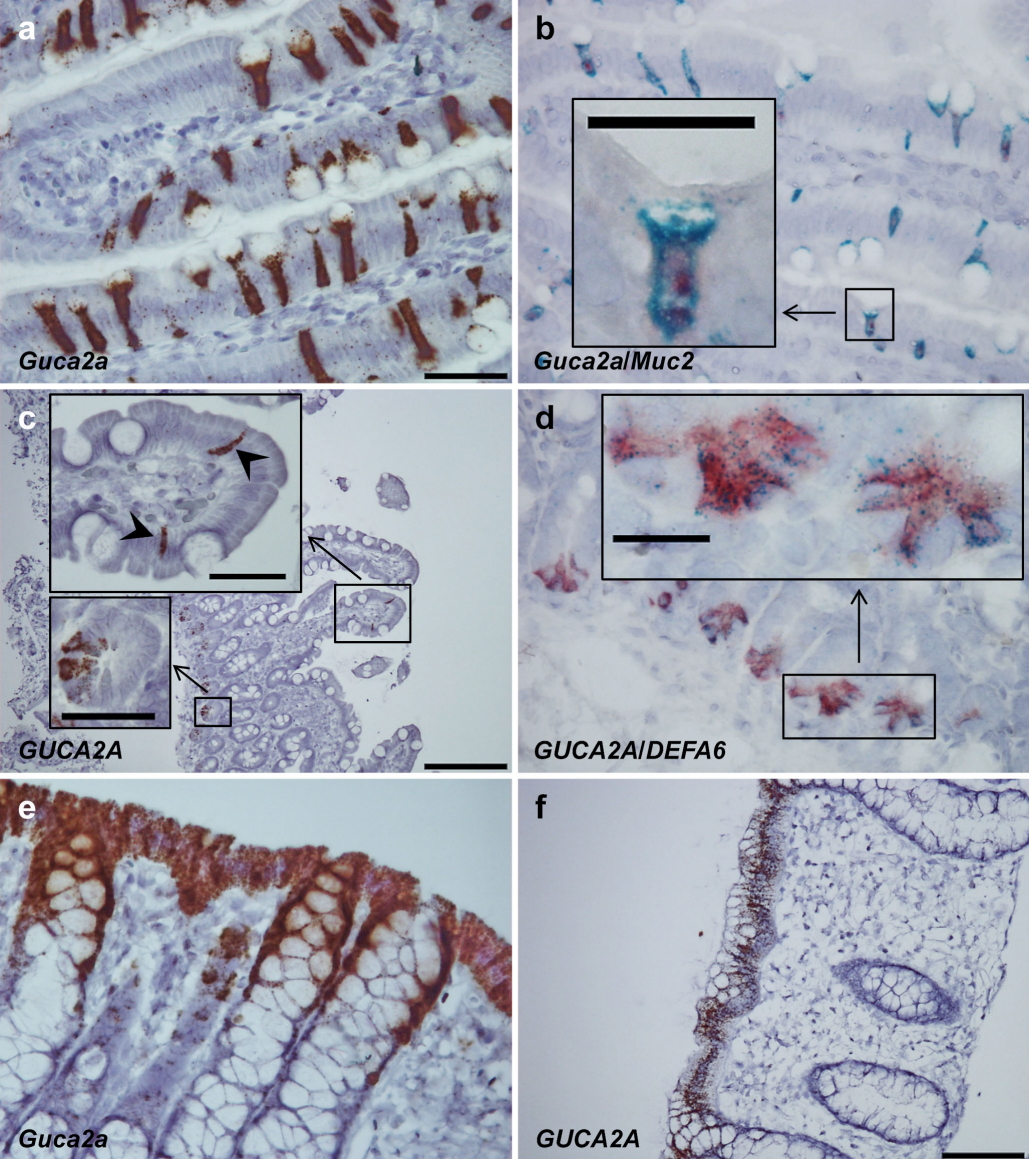
*Guca2a* and *GUCA2A* expression (guanylin) in rat and human duodenum and colon. **a** *Guca2a*-expressing cells in rat duodenal mucosa have goblet cell morphology. **b** Duplex in situ hybridization (ISH) showing that *Guca2a* (*red*) and *Muc2* (*blue*) in rat duodenal mucosa are co-expressed in goblet cells. **c** *GUCA2A*-expressing cells in human duodenum are localized basally in crypts (*small insert*) and in scattered cells with a slender shape present in the duodenal villi (*large insert*, *arrowheads*). **d** Duplex ISH for *GUCA2A* (*blue*) and *DEFA6* (*red*) demonstrates co-expression in human duodenal crypts indicating *GUCA2A* expression in Paneth cells (*large insert*). **e** In the rat colon *Guca2a* is abundantly expressed in virtually all surface epithelial cells but is not expressed from the middle to the basal parts of the crypt epithelium. **f** *GUCA2A* expression in the human colon is also localized to virtually all cells of the surface epithelium but is not expressed in the middle or basal parts of the crypt epithelium. *Bars* 20 μm (*inserts* in **b**, **d**), 50 μm (**a**, **b**, **d**, **e**, *inserts* in **c**), 100 μm (**f**), 200 μm (**c**)

In rat duodenum, *Guca2a* (GN) was localized to *Muc2*-expressing goblet cells but was not expressed in *Defa6*-positive Paneth cells (Fig. 1a, b; see also Fig. S1a–f). In human duodenum, however, *GUCA2A* was expressed in *DEFA6*-positive Paneth cells and in addition, in a few scattered epithelial cells of the duodenal villi (Fig. 1c, d). In both rat and human colonic mucosa, *Guca2a*/*GUCA2A* was expressed in the entire surface epithelium, thus being present in both goblet cells and colonocytes (Fig. 1e, f).

**Fig. 2:**
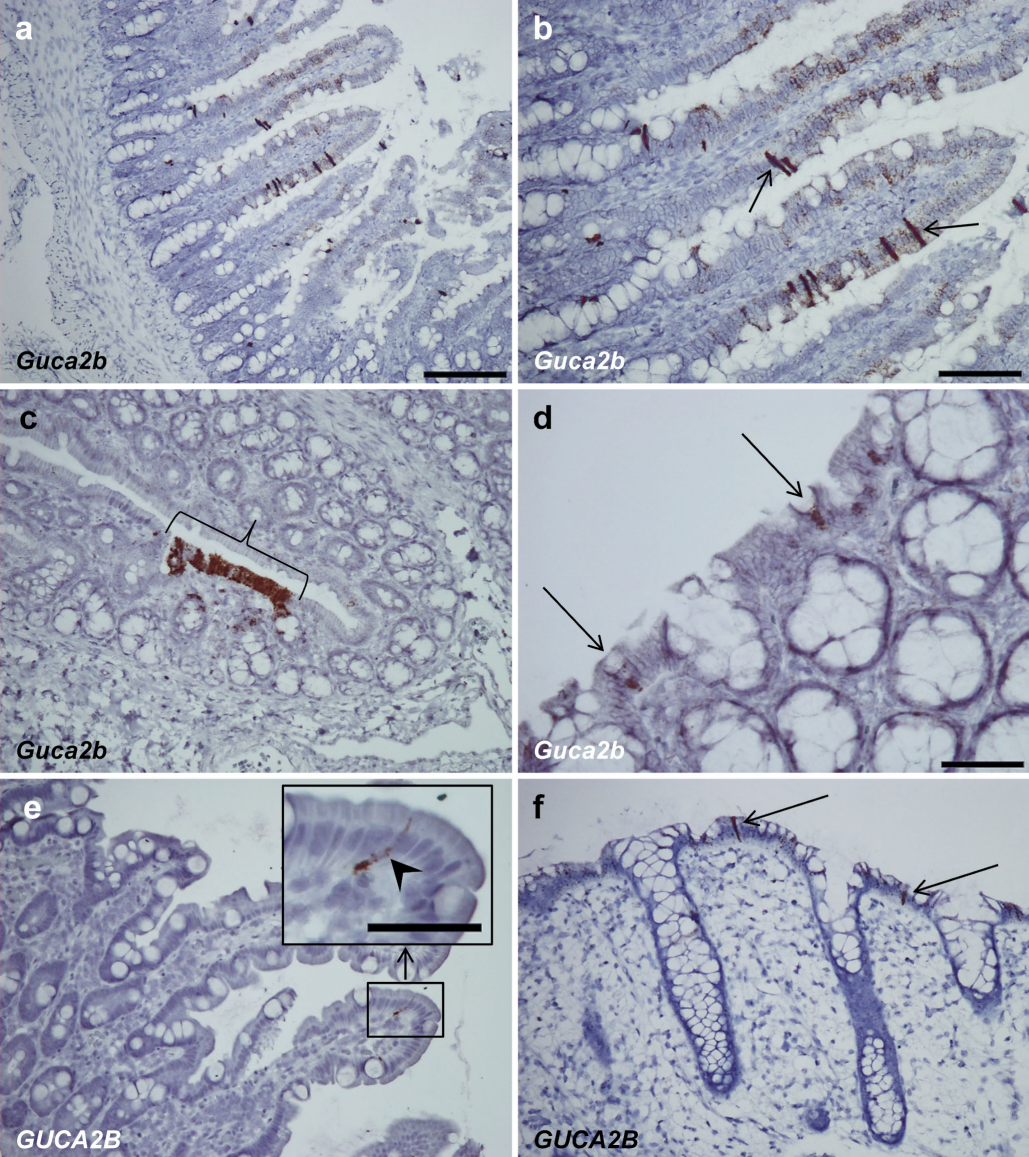
*Guca2b* and *GUCA2B* expression in rat and human duodenum and colon. **a**, **b** *Guca2b* expression in rat duodenal mucosa. Expression of *Guca2b* is strong in scattered epithelial cells. Some of these cells (**b**) display a central swelling and narrow apical and basal parts (*arrows*). Additionally, scattered small black dots can be seen in other epithelial cells, indicating that expression is not entirely specific to a distinct cell type. *Guca2b* does not seem to be expressed in crypt epithelium. **c** Focal expression of *Guca2b* in rat colonic epithelium (*bracket*). **d** Two cells (*solid arrows*), probably goblet cells, expressing *Guca2b* in rat colon. **e** Solitary *GUCA2B*-expressing epithelial cell of a human duodenal villus (*insert*, *arrowhead*). **f** Two epithelial cells expressing *GUCA2B* in human colon (*solid arrows*). Some small black dots can also be spotted in other cells of the superficial colonic epithelium. *Bars* 50 μm (**d**, *insert* in **e**), 100 μm (**b**, **c**, **e**, **f**), 200 μm (**a**)

*Guca2b* (UGN) expression in rat duodenal mucosa was widespread with mRNA being present to some degree in most cells of the duodenal villi. However, some epithelial cells had intense expression and clearly stood out (Fig. 2a, b). *Guca2b* expression was seldom present in the rat colon and seemed to be localized to clustered colonocytes and occasionally goblet cells (Fig. 2c, d). Contrary to the expression of *Guca2b* in rat duodenum, *GUCA2B* expression in human duodenum was considerably less pronounced and was limited to scattered epithelial cells within the duodenal villi (Fig. 2e). In the human colon, *GUCA2B* was mainly strongly expressed in solitary superficial epithelial cells but scattered positivity for *GUCA2B* could also be seen in other superficial epithelial cells (Fig. 2f).

**Fig. 3:**
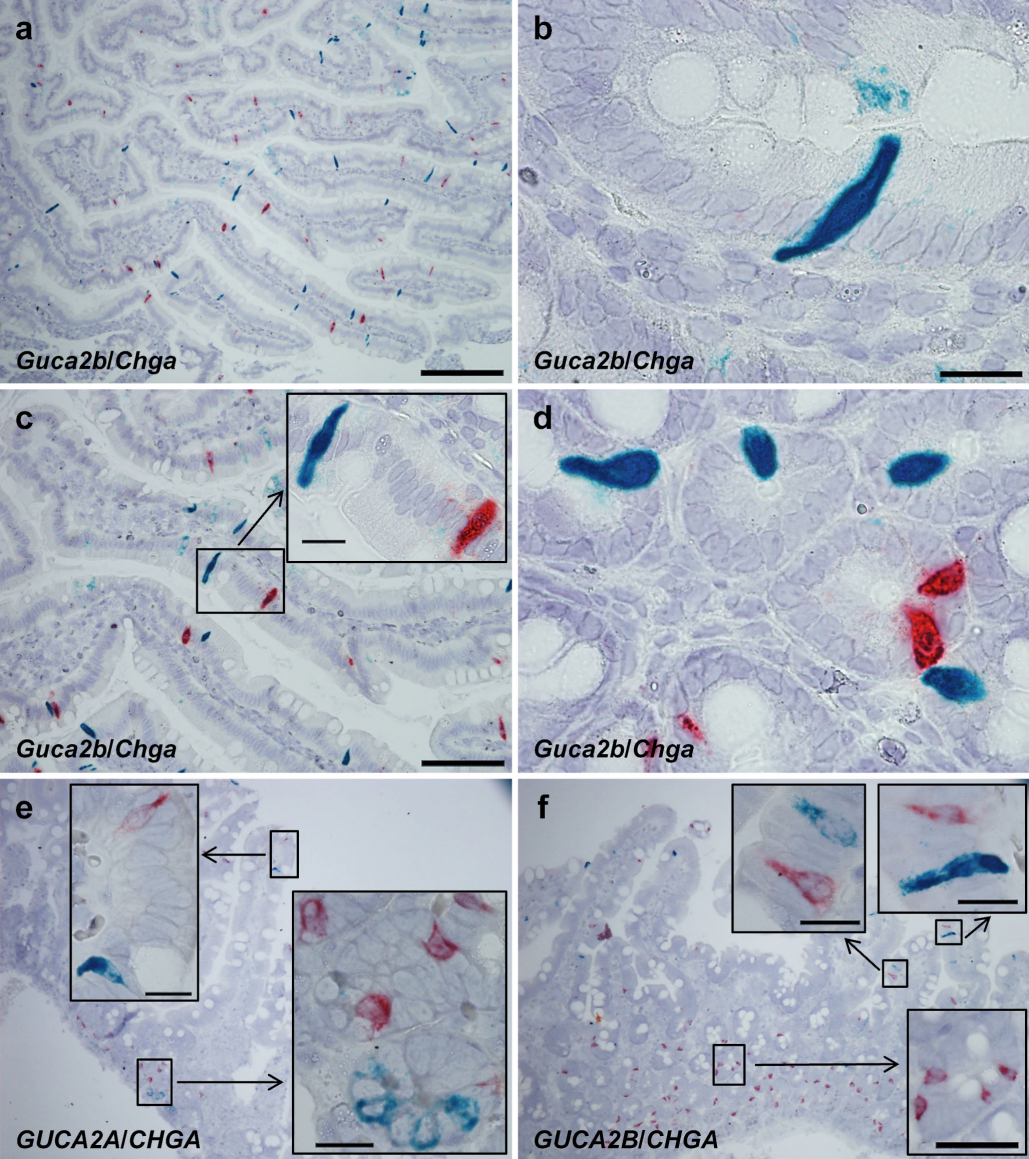
Duplex ISH of *Guca2b*/*Chga*, *GUCA2B*/*CHGA*, and *GUCA2A*/*CHGA* in rat and human duodenum. **a** Duplex ISH of *Guca2b* (*blue*) and *Chga* (*red*) showing no definitive signs of co-expression of *Chga* and *Guca2b* in rat duodenum. **b**, **c** Morphology of *Guca2b*-expressing (*blue*) epithelial cells in rat duodenal villi. The cells have a central thickening and basal and apical narrowing. Whereas **b** displays no *Chga*-expressing cells (*red*), the *Guca2b*-expressing cell (*blue*) in **c** is accompanied by an adjacent *Chga*-expressing cell (*red*). The *Chga*-expressing cell in **c** contains some scattered *blue dots* indicating some *Guca2b* expression. **d** *Guca2b* (*blue*) and *Chga* (*red*) expression in crypt cells, without overlap in the *blue* *Guca2b*-expressing cells. However, in the *red* *Chga*-expressing cells, *blue dots* indicate co-expression of *Guca2b*. **e**, **f** In human duodenum, co-expression cannot be seen for *GUCA2A* (*blue*) and *CHGA* (*red*) or for *GUCA2B* (*blue*) and *CHGA* (*red*); see *inserts*. *Bars* 20 μm (**b**, **d**, *inserts* in **c**, **e**, *upper inserts* in **f**), 50 μm (*lower insert* in **f**), 100 μm (**c**), 200 μm (**a**, **e**, **f**)

Use of duplex ISH in rat duodenum gave no convincing evidence for the co-expression of *Guca2b* and *Chga* (CgA) in strongly *Guca2b*-expressing cells (Fig. 3a–d). In human duodenum, neither *GUCA2A*- nor *GUCA2B*-positive cells co-expressed *CHGA* (CgA) (Fig. 3e, f). Serial sections of rat duodenum with conventional single-plex ISH for *Guca2b* and *Chga* also showed no overlap between strongly *Guca2b*-expressing cells and *Chga*. However, some of the *Chga*-expressing cells might also have expressed some *Guca2b* given the weak and widespread expression of *Guca2b* (Fig. S2a, b). This is also subtly indicated by duplex ISH in some *Chga*-expressing cells (Fig. 3c, d, see also Fig. S2c, d). Serial sections of the human colon also gave no indication of the co-expression of *CHGA* in strongly *GUCA2B*-expressing cells (Fig. S2e, f).

Again, we found no overlap of distinctly *Guca2b*/*GUCA2B*-expressing cells and selected tuft cell markers (*Dclk1*/*DCLK1*, *Sox9*/*SOX9*, *Gnat3*/*GNAT3*, or *Trpm5*/*TRPM5*; Fig. S3a–h) by using conventional single-plex ISH and serial sections (micrographs from human duodenum are not shown). As we questioned the sensitivity of the *Dclk1*/*DCLK1* probes, we also ran serial sections for *Guca2b*/*GUCA2B* single-plex ISH and Dclk1 IHC. Dclk1 IHC might have shown slightly better sensitivity for Dclk1 than ISH, especially in rat duodenum but again, we saw no convincing overlap between *Guca2b*/*GUCA2B* and Dclk1 (Fig. S4a–h).

As Chga/CHGA might not be expressed in all subtypes of EE cells (Cetin et al. 1989), we also examined rat and human duodenal mucosa for the expression of insulinoma-associated 1 (*Insm1*/*INSM1*), which has been shown to be expressed in EE cells and required for their differentiation (Gierl et al. 2006). In human duodenum, *INSM1* was scarcely expressed compared with *CHGA* (Fig. S5a–d). In rat duodenum, we found a slightly higher expression of *Insm1* (Fig. S6a–d); however, the expression of *Insm1* was considerably less than for both *Chga* and *Guca2b* (Fig. S6e, f). Additionally, *Insm1* was mostly expressed in crypt cells compared with *Guca2b*, which was predominantly expressed in duodenal villi (Fig. S6a–c, f).

The IHC results for GN on human duodenal and colonic sections (Fig. S7a, b) were comparable with the ISH results (Fig. 1c, f). Immunoreactivity for GN was seen in duodenal crypts consistent with localization in Paneth cells, in a few epithelial cells of the duodenal villi and in the entire superficial epithelium of the human colon. Unfortunately, because of a lack of appropriate antibodies for human and rat, we were not able to perform IHC for GN in rat or for UGN in human and rat.

For human *GUCA2A* mRNA (ISH) and GN peptide (IHC), abundance was much higher in duodenal crypt cells than in duodenal villi (Table 2). In each duodenal section, one can expect >20 positive crypt cells for every positive villous cell. Approximately half of the cells in human duodenal crypts express or synthesize *GUCA2A*/GN, whereas one would expect to find from none to two *GUCA2A*/GN-positive cells in every duodenal villus.

**Table 2:** Number of GN-positive (*IHC* immunohistochemistry) and *GUCA2A*-positive (*ISH* in situ hybridization) cells of human duodenum (crypts and villi)

*GUCA2A*/GN (entire biopsy)	Positive crypt cells	Counted crypts	Positive villous cells	Counted villi
Section 1 (IHC)	237	99	12	30
Section 2 (IHC)	>250	>100	0	43
Section 3 (ISH)	56	26	13	13
Section 4 (ISH)	116	61	12	7

In human duodenum, the average numbers of *GUCA2B*- and *CHGA*-expressing cells were 4.1 and 86.8 per visual field (200×), respectively, whereas the average numbers of *Guca2b*- and *Chga*-expressing cells in rat duodenum were 85.6 and 173.0 per visual field (200×), respectively (Table 3).

**Table 3:** Numbers of distinctly *Guca2b/GUCA2B*-expressing cells compared with *Chga/CHGA*-expressing cells in rat and human duodenum (*STD* standard deviation)

Statistic	*Guca2b * ^a^	*Chga * ^a^	*P* ^†^	*GUCA2B* ^b^	*CHGA* ^c^	*P* ^‡^
Average	85.6	173.0	0.001	4.1	86.8	0.002
STD	22.1	35.6		5.1	15.0	

Magnification 200×

† Student *t*-test equal variance

‡ Student *t*-test unequal variance.

^a^Five visual fields from two sections were examined

^b^Eight visual fields from two sections were examined

^c^Four visual fields from two sections were examined

## Discussion

The current study focuses on the cellular localization of *GUCA2A*/*Guca2a* (GN) and *GUCA2B*/*Guca2b* (UGN) mRNAs in the duodenum and colon of both human and rat. Species differences are obvious in the duodenum. In the rat, *Guca2a* is distinctly expressed in goblet cells, as confirmed by serial sectioning and duplex ISH with *Muc2*. Paneth cells in rat do not express *Guca2a*; there is an absence of *Guca2a* expression deep in duodenal crypts and of co-expression with *Defa6* in duplex ISH. In human duodenal mucosa, however, *GUCA2A* is expressed in Paneth cells, with overlapping expression with *DEFA6*. Moreover, the expression of *GUCA2A* in the human duodenal villi is limited to scattered epithelial cells and is not localized to goblet cells, consistent with previous findings in human gastrointestinal tissue (Date et al. 1996). Overall, the species differences are in concordance with previously observed differences (Cohen et al. 1995, 1998; Date et al. 1996; de Sauvage et al. 1992; Li and Goy 1993; Li et al. 1995).

The expression of *GUCA2A*/*Guca2a* in rat and human colon, however, is similar. We found colonic *GUCA2A*/*Guca2a* expression in human and rat to be present in the entire surface epithelium and, consequently, this should be present in both goblet cells and colonocytes, similar to previous findings (Date et al. 1996; Lewis et al. 1993). In rat, the release of GN into the colonic lumen has previously been determined to be 40-fold higher than in portal effluents after stimulation (Moro et al. 2000). GN acts in a luminocrine (both paracrine and autocrine) manner via activation of the GC-C receptor. The GC-C receptor is expressed throughout the intestinal epithelium of both human and rat (Krause et al. 1994; Qian et al. 2000). In human small intestine, the receptor density decreases longitudinally in the small intestine, whereas within the vertical axis of villi and crypts of the small intestine, the greatest density is observed in the basal half of the villi and in the neck region of intestinal glands (Krause et al. 1994). In the colon, the receptor is found both in superficial epithelial cells and in colonic glands (Krause et al. 1994).

In human duodenum, *GUCA2A* is expressed in Paneth cells, which secrete anti-microbial peptides and additionally, *Guca2a/GUCA2A* is expressed in mucus-secreting goblet cells in rat duodenum and in rat and human colon, a critical characteristic for epithelial defense (Van der Sluis et al. 2006). Some natriuretic peptides have antibacterial properties; however, we are only aware of one study in which GN and UGN have been tested and found to have lower antimicrobial activity (Krause et al. 2001).

We found no overlap of *GUCA2A* and *CHGA* (CgA) in human duodenal mucosa. Previous studies on mucosal sections identified GN in EE (EC and D) cells (Cetin et al. 1994; Hill et al. 1995; Ieda et al. 1998). Similarly, increased *Guca2a* expression has been seen in fluorescence-activated-sorted EC cells of *Mastomys* compared with the intestinal mucosa (Kidd et al. 2006) and serum GN is elevated in patients with carcinoid tumors (Kuhn et al. 1995). Others, on the other hand, found no co-localization of GN and CgA, serotonin, glucagon, pancreatic polypeptide, vasoactive intestinal polypeptide, or somatostatin (Date et al. 1996). Previously, most epithelial cell lineages have been postulated to express GN mRNA (Cohen et al. 1998). Our findings in the colon of both human and rat indicate that GN is not entirely specific to a particular cell type, as the entire colonic epithelium seems to express *GUCA2A*/*Guca2a*. In any case, the current findings are in harmony with most previous IHC or ISH studies regarding GN and RNA Scope ISH technology is also extremely robust with high specificity and sensitivity, given a successful probe (Sordal et al. 2013).

*Guca2b* was strongly expressed in distinct cells of the rat duodenum and was additionally widespread, although weak expression was seen in other epithelial cells of the duodenal villi. Thus, the possibility that some EC cells also express *Guca2b* is high. Indeed, we demonstrated this with duplex ISH in rat duodenum in which subtle *Guca2b* expression could be detected in *Chga*-expressing cells. This is partly consistent with previous findings in rat jejunum in which most serotonin-positive cells are UGN-positive and vice versa, although a subset of positive cells is only positive for each peptide (Perkins et al. 1997). In the same study, no co-localization of cholecystokinin (CCK)-synthesizing I-cells and UGN was seen. In our study, the epithelial cells that displayed intense *Guca2b* expression clearly stood out from the rest of the epithelial cell population. These cells did not display *Chga* expression and are therefore probably not EC cells (Portela-Gomes et al. 1997). Thus, these predominantly *Guca2b*-expressing cells might be a different and possibly not yet characterized, cell type. In rat colon, *Guca2b* expression is patchy in what appears to be colonocytes. *Guca2b* is also expressed in some rat colonic cells with a goblet cell appearance, further indicating that *Guca2b* expression is not entirely cell-specific and might be induced in multiple cell types.

*GUCA2B* expression seems to be more specific in humans. In the duodenum, expression is limited to a small number of solitary epithelial cells of the duodenal villi, whereas colonic *GUCA2B* expression is mainly localized to distinct superficial cells. These cells do not seem to express *CHGA*; however, this was only determined by the interpretation of serial sections. Whether these cells also represent a specific cell type as suggested for rat duodenum remains to be clarified. Two previous studies have indicated that human UGN is synthesized in EE cells of the colon (Hess et al. 1995) and in D cells in the stomach (Mägert et al. 1998). These investigations and our study are, to the best of our knowledge, the only ones to show cell-specific cellular localization of UGN/*GUCA2B*; we are not aware of other studies showing cell-specific localization of human UGN/*GUCA2B* in the duodenum or small intestine.

The loss of GC-C signaling has been shown to disrupt satiety and food intake decreases after UGN injection in mice (Valentino et al. 2011). Thus, *GUCA2B*/*Guca2b*-expressing cells in the small intestine probably have sensory properties and might be of importance in the pathogenesis of obesity. Additionally, circulating GN and UGN regulate natriuresis (Mueller and Dieplinger 2012). From the morphology of many of the *Guca2b* cells presented in this paper, we speculate that these cells might be tuft cells. Tuft cells, also referred to as brush or caveolated cells (Kokrashvili et al. 2009), are scattered epithelial cells, having been recognized as a distinct cell type in rat tracheal epithelium in 1956 (Rhodin and Dalhamn 1956). Morphologically, they are characterized by narrow apical and basal parts and a swelling of the nuclear portion, with apical microvilli (Sato 2007). Proposed functions are absorptive, secretory and chemoreceptive (Sato 2007). Transient receptor potential cation channel (*Trpm5*)-expressing tuft cells in mice have been proposed as a cellular source for UGN (Kokrashvili et al. 2009). These cells do not express CgA or CgB or any associated peptide or amine (Kokrashvili et al. 2009). Several tuft cell markers have been proposed, including calmodulin-dependent protein kinase-like-1 (*Dclk1*), *Trpm5*, α-gustducin (*Gnat3*) and sex-determining region Y-box (*Sox9*; Gerbe et al. 2012). *Dlck1* is categorized as a structural marker, whereas *Gnat3* and *Trpm5* are termed taste-cell-related markers (Gerbe et al. 2012). *Sox9* is not entirely tuft-cell-specific but is also expressed in Paneth cells and is essential for their differentiation (Gerbe et al. 2011). In serial human and rat duodenal sections stained by using the above-mentioned tuft cell markers, we have, however, been unable to determine co-expression with *GUCA2B*/*Guca2b*-expressing cells. The expression of two of these proposed tuft cell markers, namely *DCLK1*/*Dclk1* and *GNAT3*/*Gnat3*, is scarce (especially in human intestine) and expression levels are significantly lower than those for *GUCA2B*/*Guca2b*. The Dclk1 IHC signal seems to show better sensitivity for Dclk1 than in ISH, suggesting that the probes for *DCLK1*/*Dclk1* are not optimal or that expression is temporal and varying. *SOX9*/*Sox9* is also rarely expressed in duodenal villi but pronounced expression has been noted deep in the crypts, consistent with its expression in Paneth cells. *TRPM5*/*Trpm5* is expressed both in solitary epithelial cells of the duodenal villi and at the base of the crypts. Strongly *GUCA2B*/*Guca2b*-expressing cells thus probably represent a specific cell type but in the absence of a reliable and consistent tuft cell marker, the assignment of UGN to the tuft cell might be difficult.

One limitation of this study is the inability to perform IHC for rat GN and both human and rat UGN because of a lack of good, commercially available antibodies. Another limitation is that CgA is questioned as a universal marker for EE cells (Cetin et al. 1989). The latter study shows that D-, CCK- and motilin (Mo)-positive cells display little CgA immunoreactivity and that significant inter- and intra-species variation is present. However, other studies have also demonstrated some CgA immunoreactivity in D cell (Portela-Gomes et al. 1997). Other EE cells and the EC cell, in particular, stain for CgA (Portela-Gomes et al. 1997). IHC might also have limitations in this setting, as different antisera raised against different CgA epitopes display variable immunoreactivity against CgA and the D cell might also be positive when examined with the appropriate antibody (Norlén et al. 2001). Thus, ISH might be advantageous for the detection of *CHGA*/*Chga* expression in this setting, as the mRNA would not be exposed to the posttranslational modifications or degradation of the CgA protein. We also examined the expression of another proposed marker for EE cells, namely *INSM1*/*Insm1* (Gierl et al. 2006) but found that expression is very weak compared with that for *CHGA*/*Chga*. In view of the difficulties in performing IHC, the ISH technology used in this study is an excellent substitute and we also believe that it is advantageous given its high sensitivity and specificity (Sordal et al. 2013).

In conclusion, by use of a highly sensitive and specific ISH method, we found that a similar pattern of cellular *GUCA2A*/*Guca2a* mRNA expression is present in human and rat colon (goblet cells and colonocytes). However, distinct interspecies differences are apparent in duodenal *GUCA2A*/*Guca2a* mRNA and in duodenal and colonic *GUCA2B*/*Guca2b* mRNA expression. In the human duodenum, *GUCA2A* is expressed in Paneth cells and in a few solitary villous epithelial cells, whereas in the rat duodenum, *Guca2a* is abundantly expressed in goblet cells. We found no evidence for the co-expression of *GUCA2A* and *GUCA2B* with *CHGA* in human intestine. Strongly *Guca2b*-expressing cells of rat duodenum also do not co-express *Chga*. Thus, EC cells are unlikely to be the main source for GN or UGN in humans and for UGN in rat. Some EE cells, at least in rat, are probably a minor source for UGN. This is also consistent with previous findings. However, the tuft cell, in particular, should be further examined, as UGN might be a major secretory product thereof. Furthermore, this is, to the best of our knowledge, the first study to show the expression of *GUCA2B* at the cellular level in human duodenum. Additional studies are needed to determine more precisely the cellular sources of, especially, UGN in humans and other species and of GN in human duodenum. Quantification of both *GUCA2A*- and *GUCA2B*-expressing cells should be conducted in the various small intestinal segments of humans.

## Electronic supplementary material

Below is the link to the electronic supplementary material.

Fig. S1 *Guca2a*, *Muc2* and *Defa6* expression in rat duodenum. **a** *Guca2a* expression in goblet cells in rat duodenum. **b** *Muc2* expression in rat duodenum. **c**, **d** Serial sections of *Guca2a* and *Muc2* expression in rat duodenum showing that the morphology of *Guca2a*- and *Muc2*-expressing cells is that of goblet cells. **e** *Defa6* expression in rat duodenum in basal crypts consistent with the localization of Paneth cells. **f** Duplex ISH of *Guca2a* (*blue*) and *Defa6* (*red*) showing no expression of *Guca2a* in *Defa6*-expressing cells in basal crypts (*arrowhead*, *insert*). *Bars* 20 μm (*insert* in **f**), 50 μm (**c**, **d**), 100 μm (**f**), 200 μm (**a**, **b**, **e**) (GIF 635 kb)

High resolution image file (TIF 20 mb)

Fig. S2 *Guca2b* and *Chga* expression in rat duodenum and *GUCA2B* and *CHGA* expression in human colon. **a**, **b** Serial sections showing *Guca2b* and *Chga* expression in rat duodenum with no definite signs of overlapping expression in strongly *Guca2b*-expressing cells (*solid arrows*) and *Chga*-expressing cells (*dotted arrows*). However, *Guca2b* expression is also present to a lesser degree in other epithelial cells (*multiple black dots*) substantiating the possibility of expression of *Guca2b* in *Chga*-expressing cells. **c**, **d** Duplex ISH of *Guca2b* and *Chga* in rat duodenum showing no co-expression of *Chga* (*red*) in cells strongly expressing *Guca2b* (*blue*), whereas some expression of *Guca2b* is present in *Chga*-expressing cells, indicated by *scattered blue dots* within the strongly *red* *Chga*-expressing cells. **e**, **f** Serial sections of *GUCA2B* and *CHGA* in human colon likewise showing that the *GUCA2B*-expressing cells (*solid arrows*) in the superficial epithelium do not overlap with the *CHGA*-expressing cell (*dotted arrow*). *Bars* 20 μm (**c**, **d**), 100 μm (**a**, **b**, **e**, **f**) (GIF 628 kb)

High resolution image file (TIF 21.6 mb)

Fig. S3 *Guca2b* and tuft cell markers. **a–h** Serial sections of *Guca2b* (**a**, **c**, **e**, **g**) and tuft cell markers *Dclk1* (**b**), *Sox9* (**d**), *Gnat3* (**f**) and *Trpm5* (**h**) in rat duodenum. Distinctly *Guca2b*-expressing cells show no overlap with scarcely present *Dclk1*-, *Gnat3*-, *Sox9*-, or *Trpm5*-expressing cells. *Bars* 50 μm (*inserts* in **a–h**), 200 μm (**a–h**) (GIF 732 kb)

High resolution image file (TIF 23.8 mb)

Fig. S4 *Guca2b*/*GUCA2B* (ISH) and Dclk1 (IHC). **a–d** Serial sections of *GUCA2B* (ISH) and Dclk1 (IHC) in human duodenum showing no definitive overlap in adjacent sections. **e–h** Serial sections of *Guca2b* (ISH) and Dclk1 (IHC) showing no overlap between cells with strong *Guca2b* expression and the location of Dclk1-positive cells in the adjacent section. *Bars* 50 μm (**c**, **d**, **g**, **h**), 100 μm (**a**, **b**, **e**, **f**) (GIF 714 kb)

High resolution image file (TIF 8.38 mb)

Fig. S5 Comparison of *INSM1* and *CHGA* expression in human duodenum. **a–d** Expression of *INSM1* is barely detectable (**a**, **c**, **d**), whereas adjacent section (**b**) of **a** shows intense expression of *CHGA*. *Bars* 50 μm (**d**), 100 μm (**c**), 500 μm (**a**, **b**) (GIF 399 kb)

High resolution image file (TIF 4.64 mb)

Fig. S6 Comparison of *Insm1*, *Chga* and *Guca2b* expression in rat duodenum. **a–d** *Insm1* expression is scarce (**a**, **b**) and mainly localized in duodenal crypts (**b**, **c**). Almost no expression can be seen in villous epithelial cells (**d**). The expression of both *Chga* (**e**) and *Guca2b* (**f**) is stronger and more widespread compared with that for *Insm1*. Additionally, *Guca2b* is mainly expressed in cells of duodenal villi. *Bars* 50 μm (**c**, **d**), 100 μm (**b**), 200 μm (**a**, **e**, **f**) (GIF 660 kb)

High resolution image file (TIF 8.06 mb)

Fig. S7 GN (IHC) in human duodenum and colon. **a** In the duodenum, GN is localized to crypts and occasional epithelial cells of the duodenal villi (*insert*, *arrowhead*). **b** In the colon, strong GN immunoreactivity is seen in the entire superficial epithelial lining. The results of GN IHC are identical to those of *GUCA2A* ISH. *Bars* 100 μm (**a**, **b**), 50 μm (*insert* in **a**) (GIF 225 kb)

High resolution image file (TIF 2.43 mb)
