# Supplementary figures and images for: Cellular localization of guanylin and uroguanylin mRNAs in human and rat duodenal and colonic mucosa
Source: Cell Tissue Res. 2016 Apr 5;365:331–41. doi: 10.1007/s00441-016-2393-y (PMC4943973; doi:10.1007/s00441-016-2393-y)

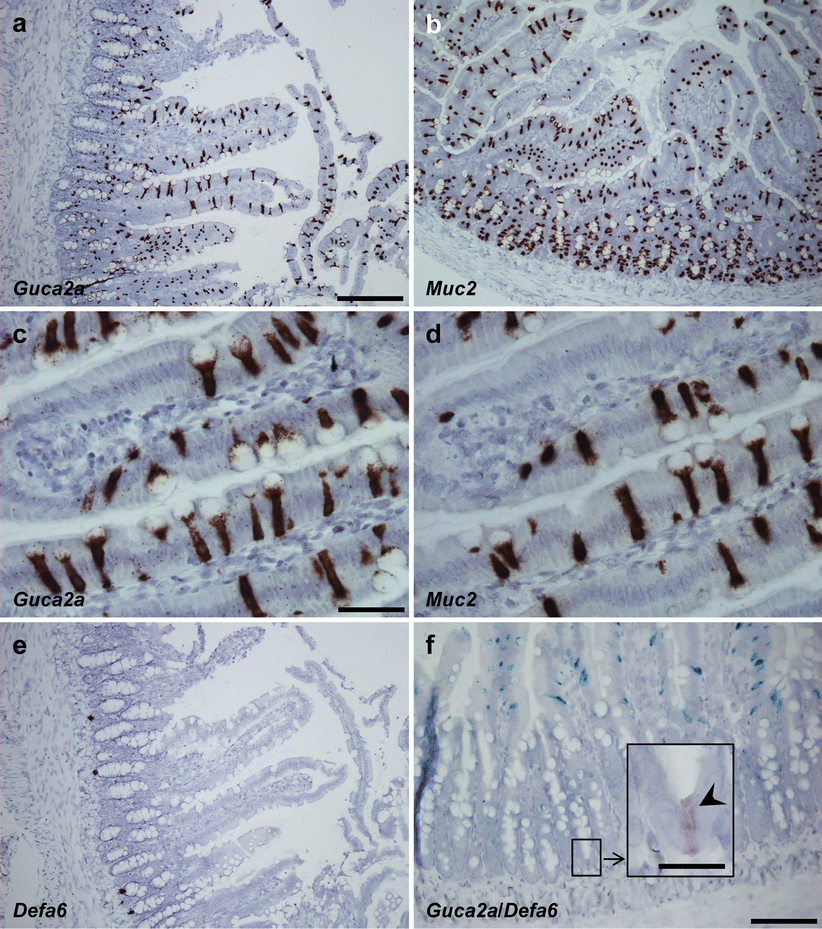

Supplement: Supplementary file 1 — Guca2a, Muc2 and Defa6 expression in rat duodenum. a Guca2a expression in goblet cells in rat duodenum. b Muc2 expression in rat duodenum. c, d Serial sections of Guca2a and Muc2 expression in rat duodenum showing that the morphology of Guca2a- and Muc2-expressing cells is that of goblet cells. e Defa6 expression in rat duodenum in basal crypts consistent with the localization of Paneth cells. f Duplex ISH of Guca2a (blue) and Defa6 (red) showing no expression of Guca2a in Defa6-expressing cells in basal crypts (arrowhead, insert). Bars 20 μm (insert in f), 50 μm (c, d), 100 μm (f), 200 μm (a, b, e) (GIF 635 kb) [file 441_2016_2393_Fig4_ESM.gif]

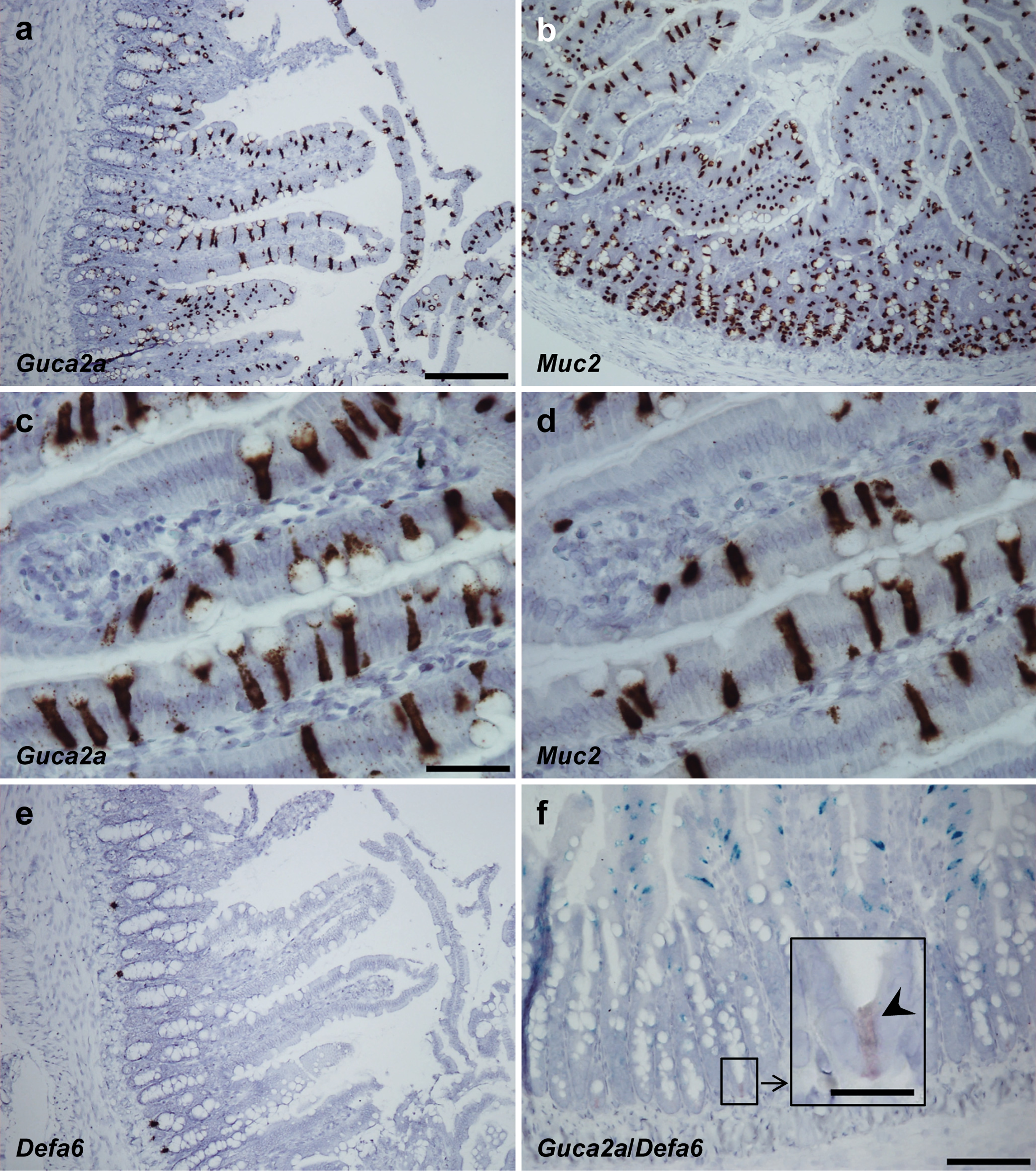

Supplement: Supplementary file 2 — High resolution image file (TIF 20 mb) [file 441_2016_2393_MOESM1_ESM.tif]

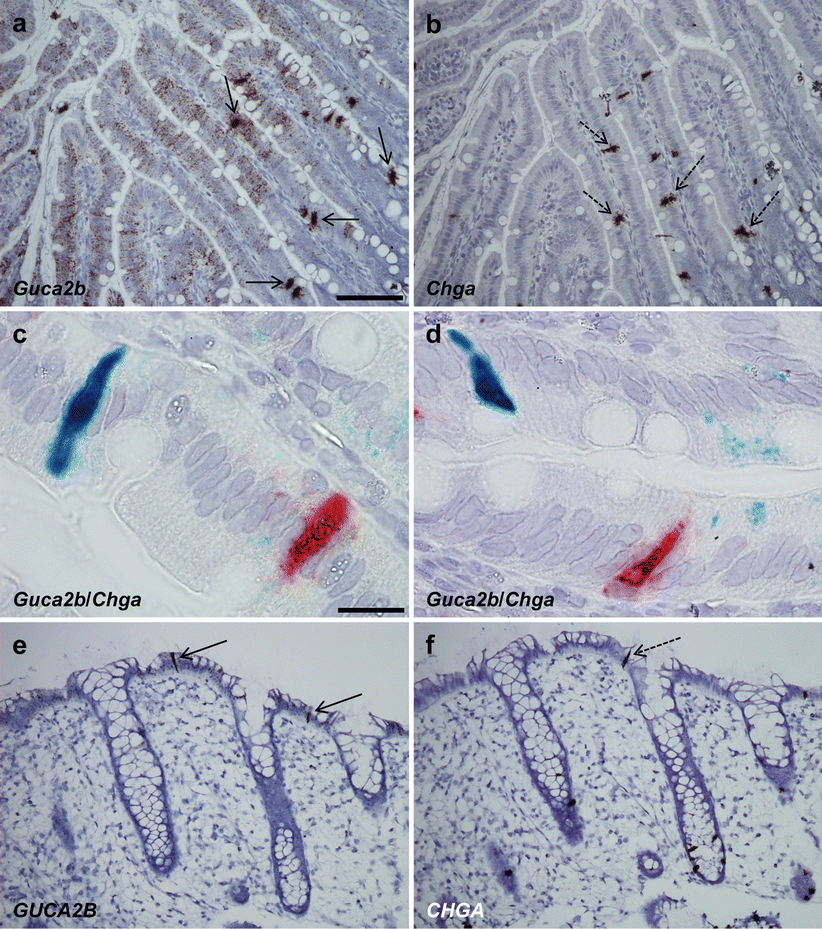

Supplement: Supplementary file 3 — Guca2b and Chga expression in rat duodenum and GUCA2B and CHGA expression in human colon. a, b Serial sections showing Guca2b and Chga expression in rat duodenum with no definite signs of overlapping expression in strongly Guca2b-expressing cells (solid arrows) and Chga-expressing cells (dotted arrows). However, Guca2b expression is also present to a lesser degree in other epithelial cells (multiple black dots) substantiating the possibility of expression of Guca2b in Chga-expressing cells. c, d Duplex ISH of Guca2b and Chga in rat duodenum showing no co-expression of Chga (red) in cells strongly expressing Guca2b (blue), whereas some expression of Guca2b is present in Chga-expressing cells, indicated by scattered blue dots within the strongly red Chga-expressing cells. e, f Serial sections of GUCA2B and CHGA in human colon likewise showing that the GUCA2B-expressing cells (solid arrows) in the superficial epithelium do not overlap with the CHGA-expressing cell (dotted arrow). Bars 20 μm (c, d), 100 μm (a, b, e, f) (GIF 628 kb) [file 441_2016_2393_Fig5_ESM.gif]

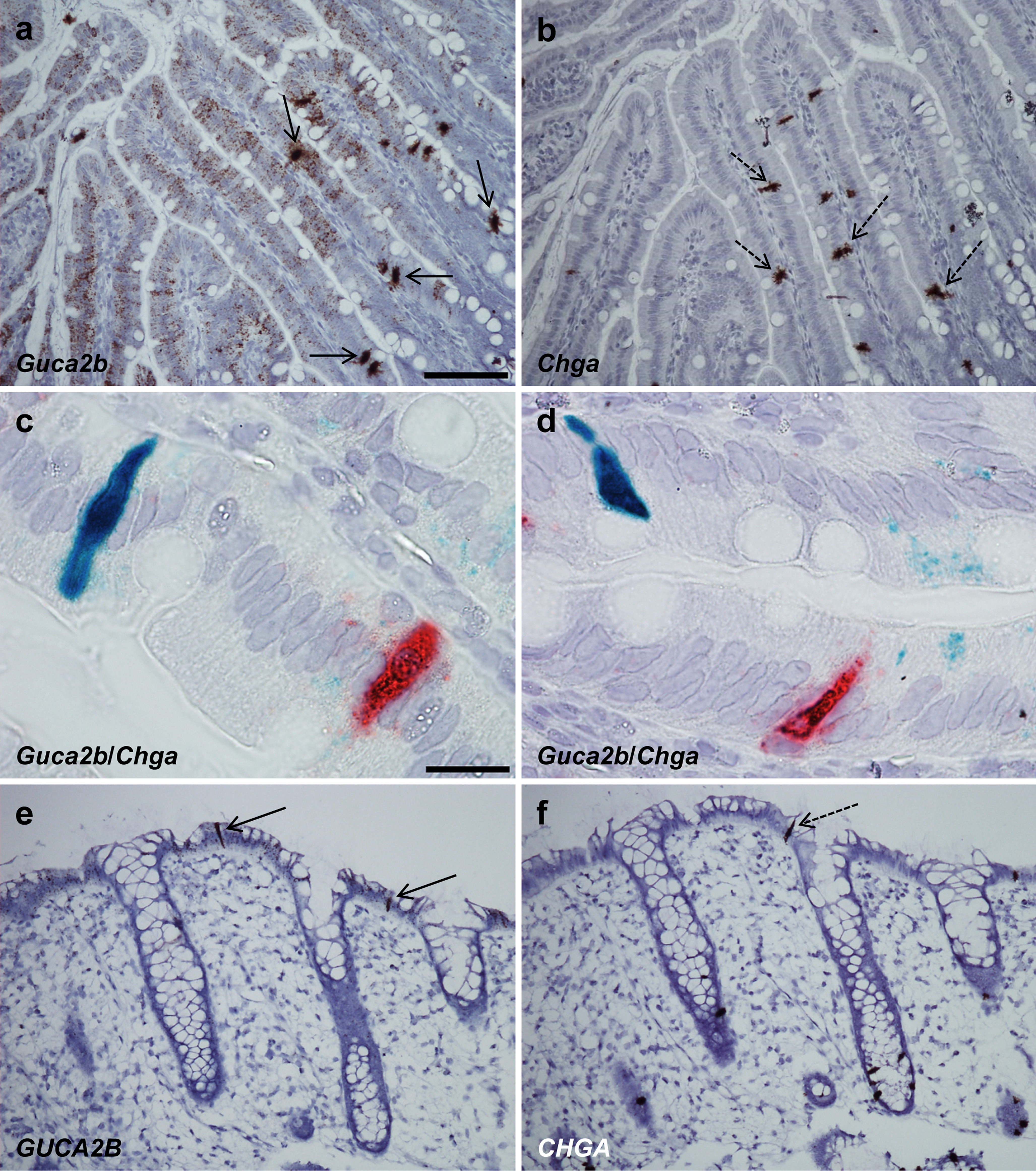

Supplement: Supplementary file 4 — High resolution image file (TIF 21.6 mb) [file 441_2016_2393_MOESM2_ESM.tif]

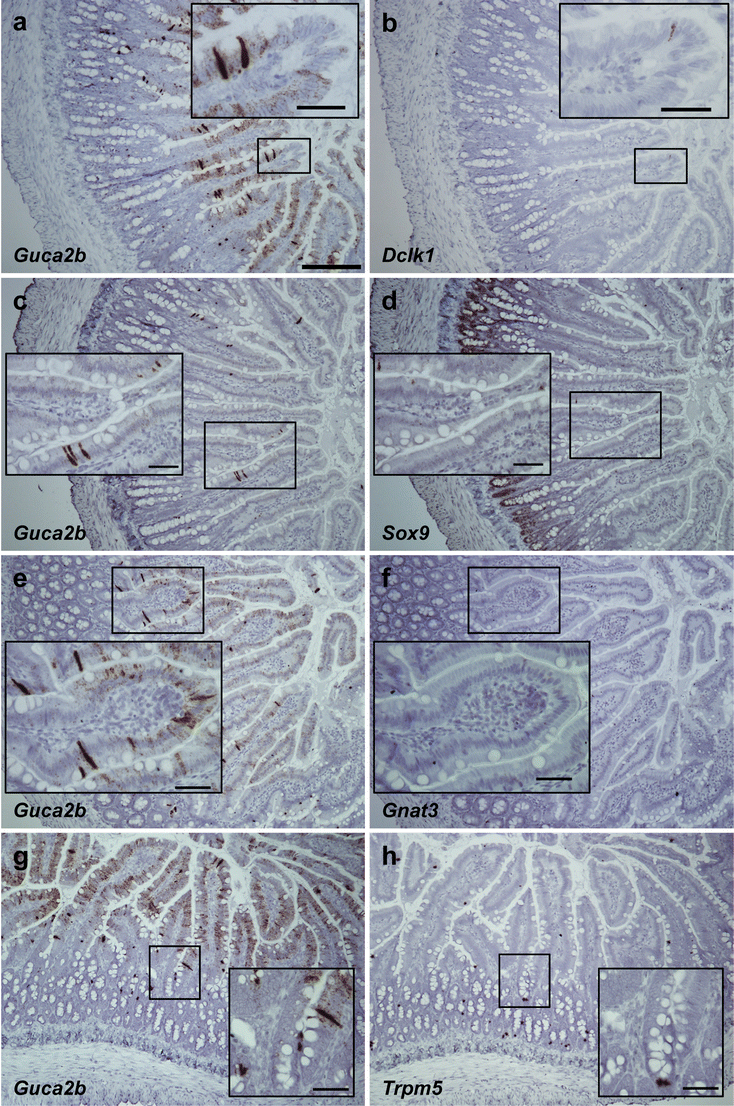

Supplement: Supplementary file 5 — Guca2b and tuft cell markers. a–h Serial sections of Guca2b (a, c, e, g) and tuft cell markers Dclk1 (b), Sox9 (d), Gnat3 (f) and Trpm5 (h) in rat duodenum. Distinctly Guca2b-expressing cells show no overlap with scarcely present Dclk1-, Gnat3-, Sox9-, or Trpm5-expressing cells. Bars 50 μm (inserts in a–h), 200 μm (a–h) (GIF 732 kb) [file 441_2016_2393_Fig6_ESM.gif]

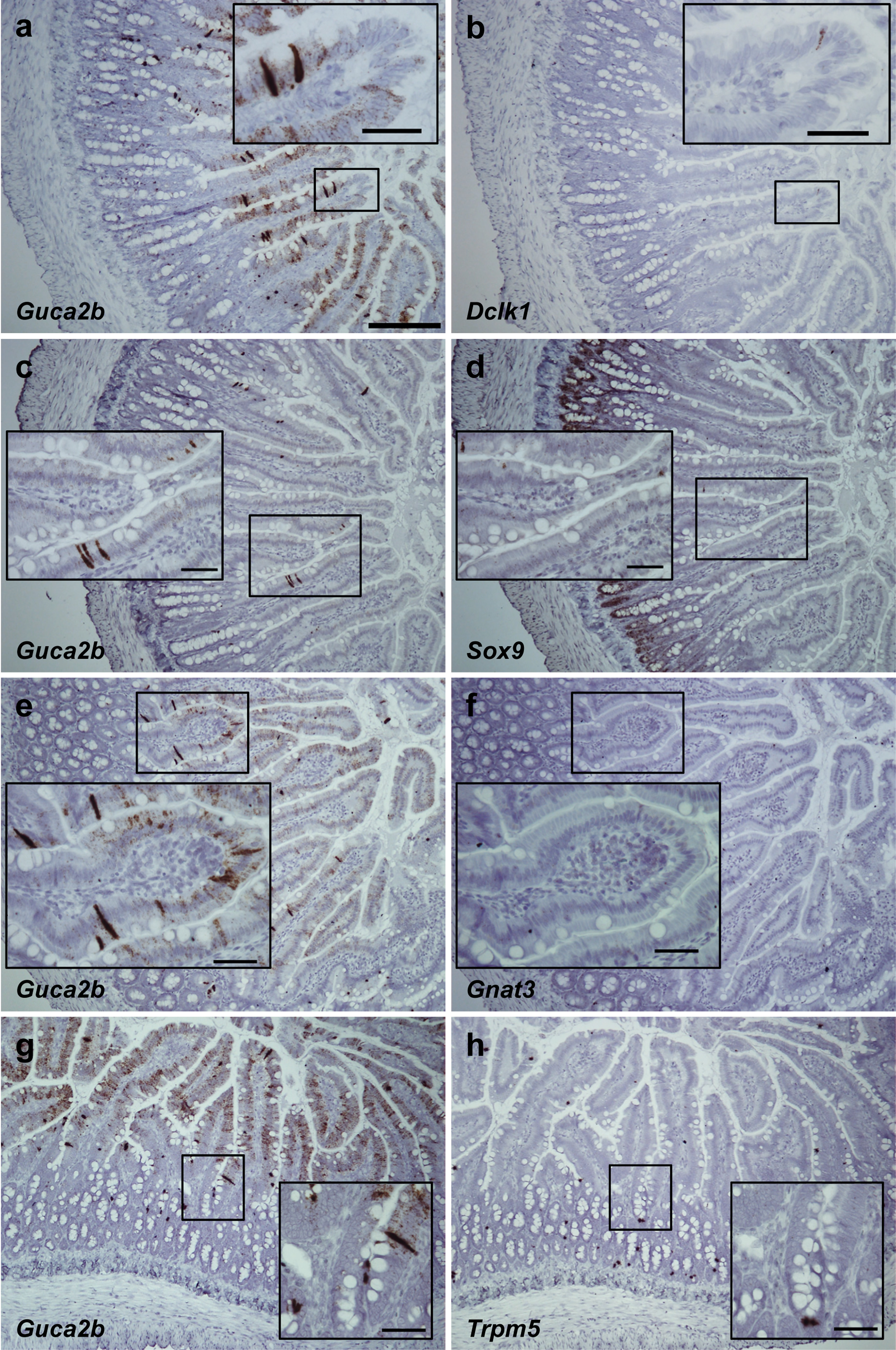

Supplement: Supplementary file 6 — High resolution image file (TIF 23.8 mb) [file 441_2016_2393_MOESM3_ESM.tif]

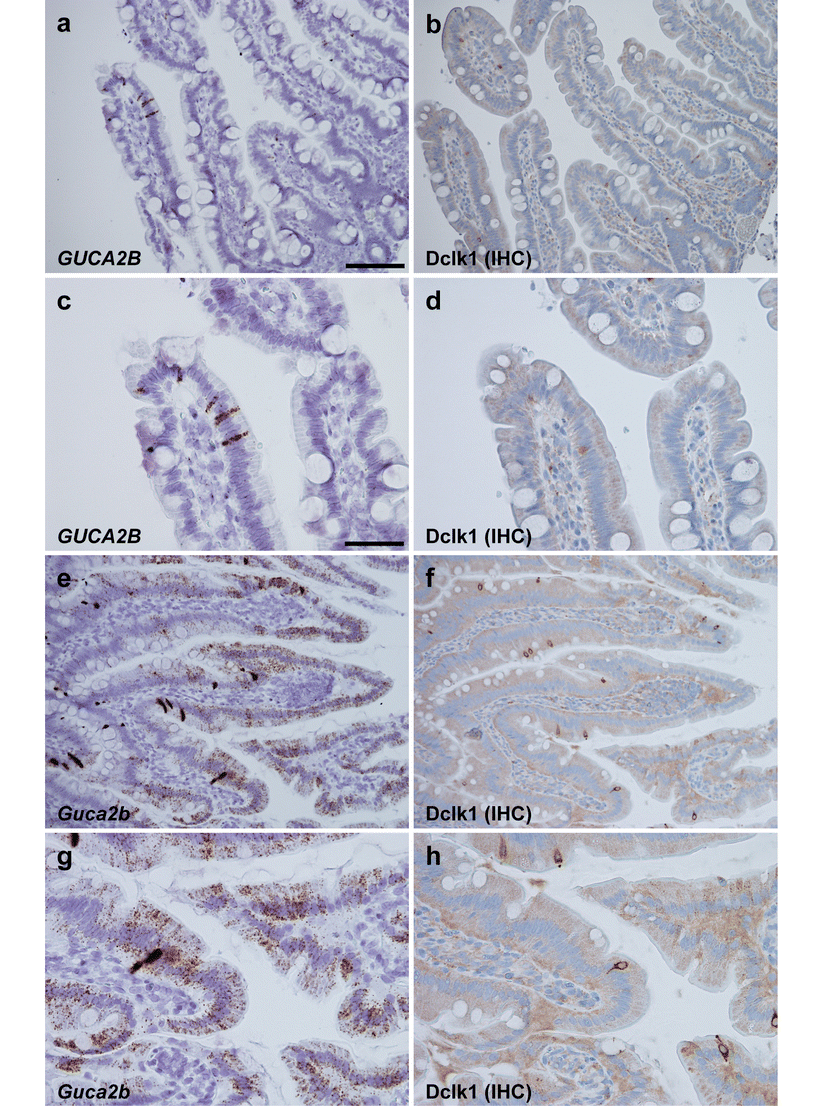

Supplement: Supplementary file 7 — Guca2b/GUCA2B (ISH) and Dclk1 (IHC). a–d Serial sections of GUCA2B (ISH) and Dclk1 (IHC) in human duodenum showing no definitive overlap in adjacent sections. e–h Serial sections of Guca2b (ISH) and Dclk1 (IHC) showing no overlap between cells with strong Guca2b expression and the location of Dclk1-positive cells in the adjacent section. Bars 50 μm (c, d, g, h), 100 μm (a, b, e, f) (GIF 714 kb) [file 441_2016_2393_Fig7_ESM.gif]

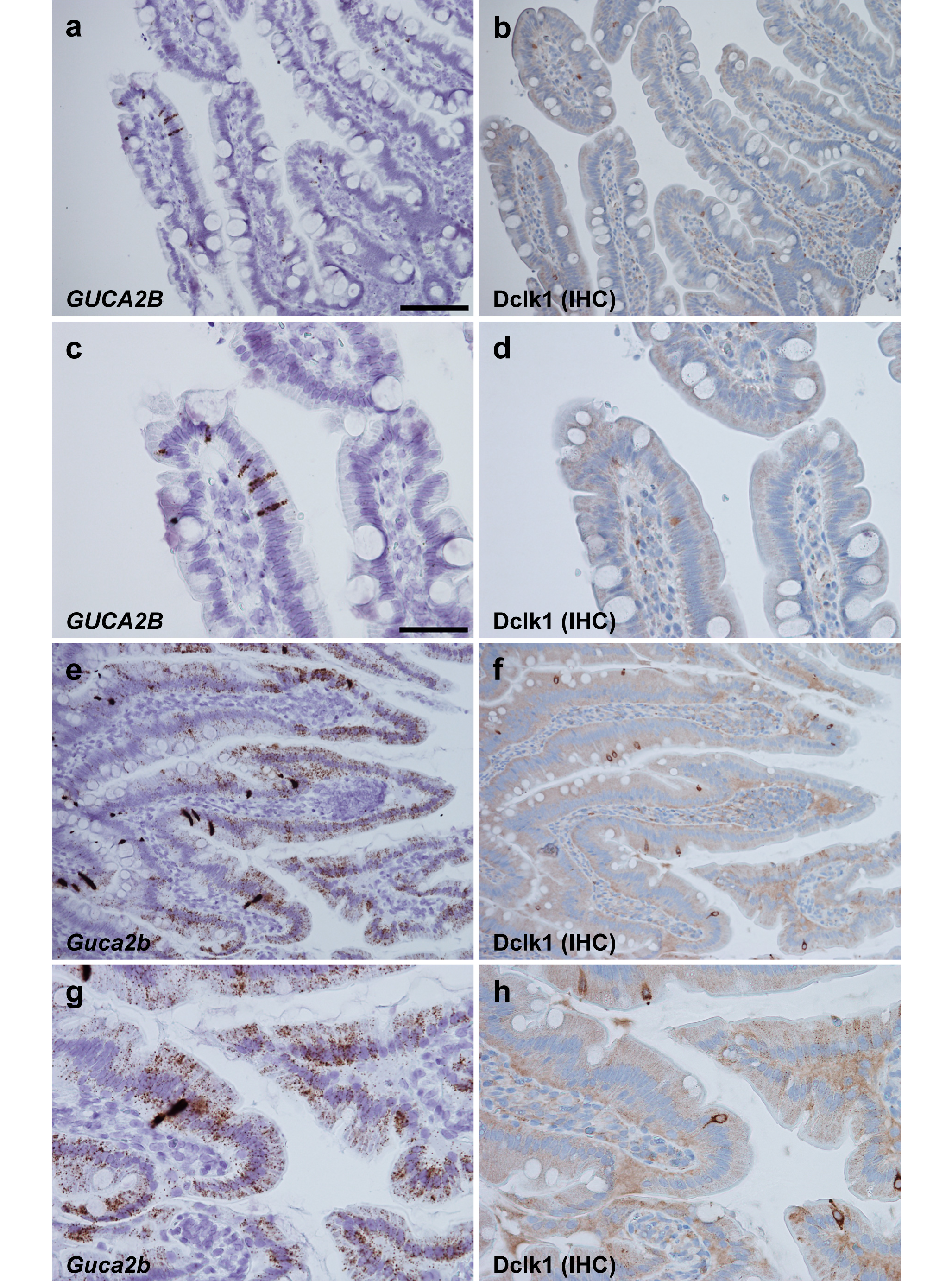

Supplement: Supplementary file 8 — High resolution image file (TIF 8.38 mb) [file 441_2016_2393_MOESM4_ESM.tif]

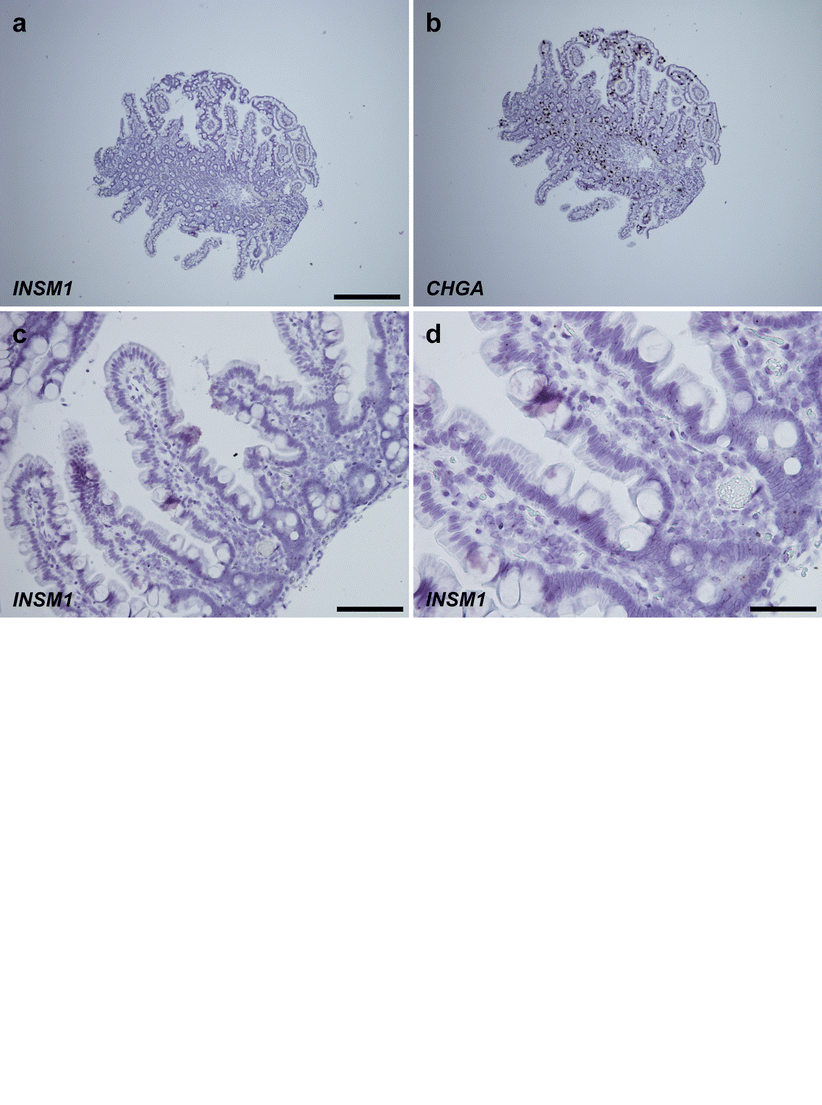

Supplement: Supplementary file 9 — Comparison of INSM1 and CHGA expression in human duodenum. a–d Expression of INSM1 is barely detectable (a, c, d), whereas adjacent section (b) of a shows intense expression of CHGA. Bars 50 μm (d), 100 μm (c), 500 μm (a, b) (GIF 399 kb) [file 441_2016_2393_Fig8_ESM.gif]

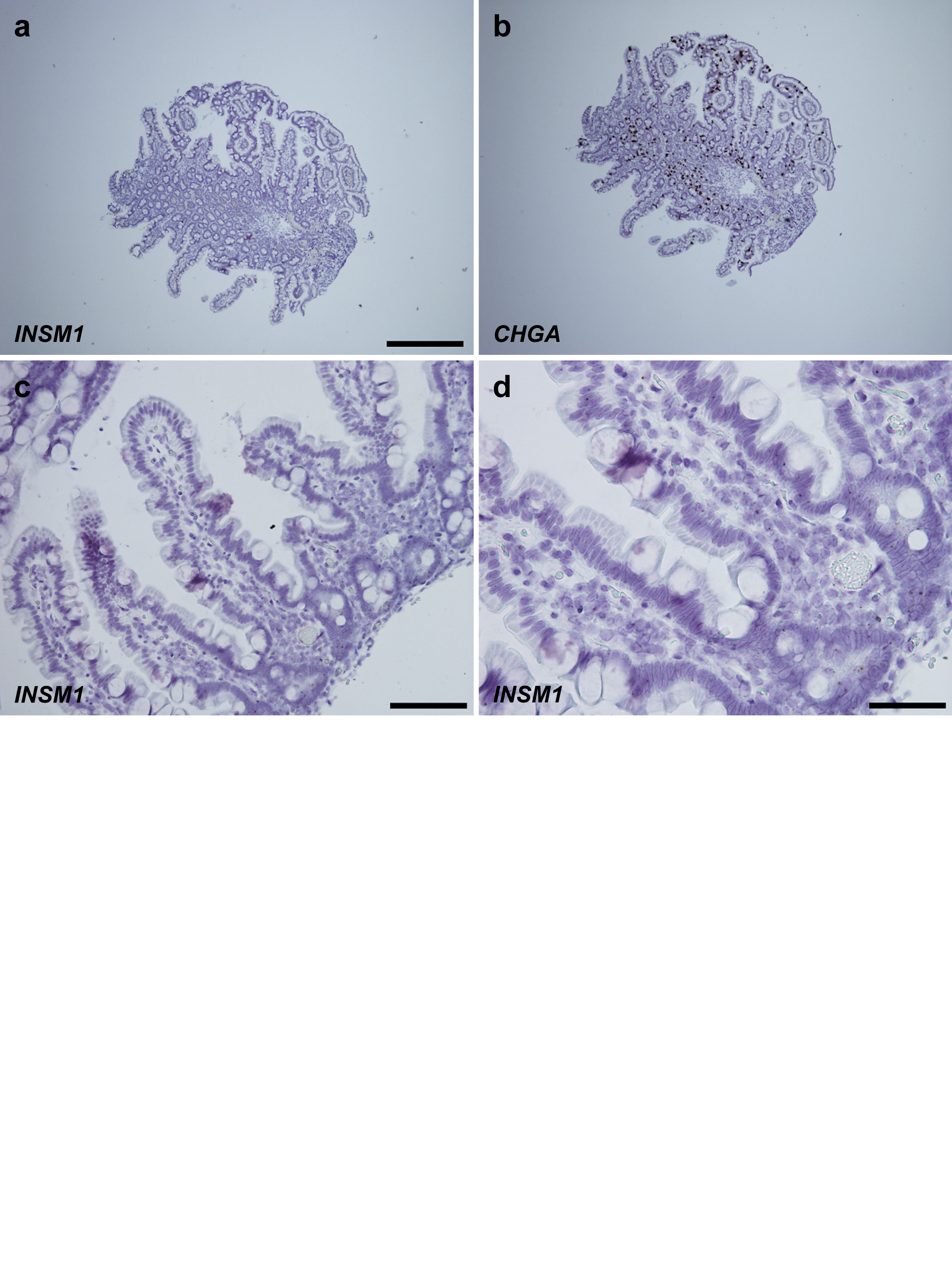

Supplement: Supplementary file 10 — High resolution image file (TIF 4.64 mb) [file 441_2016_2393_MOESM5_ESM.tif]

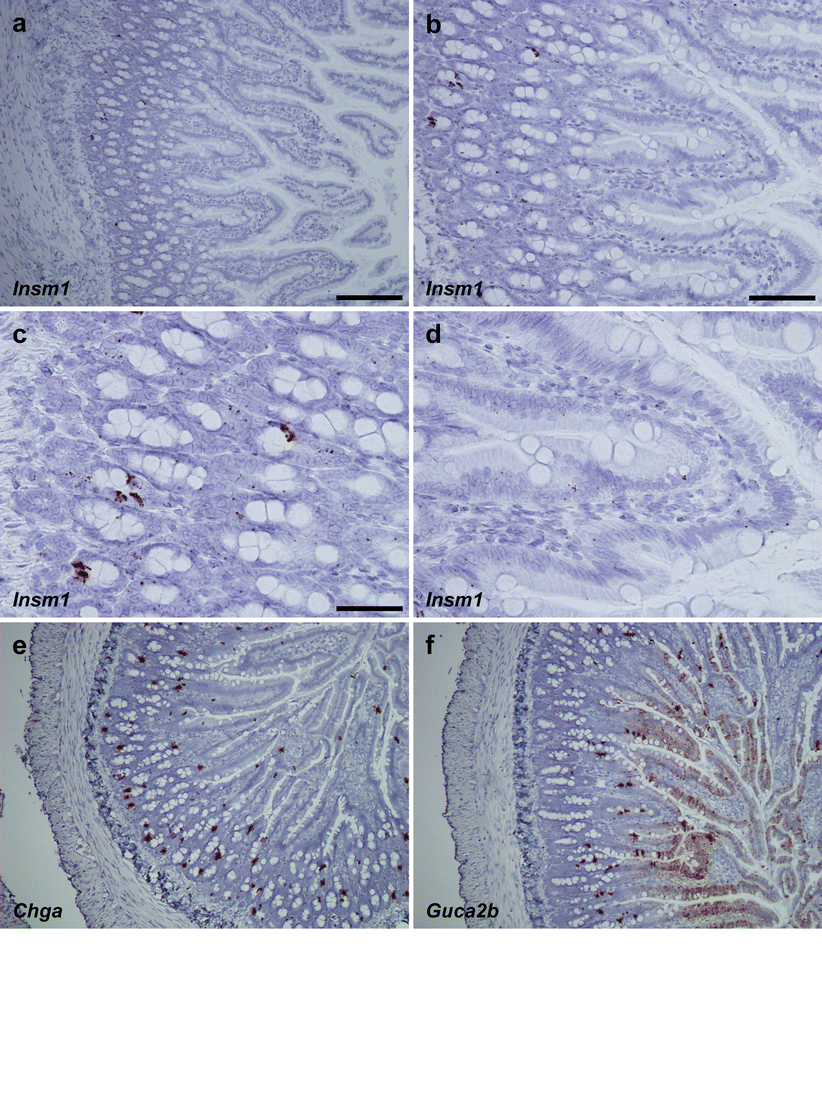

Supplement: Supplementary file 11 — Comparison of Insm1, Chga and Guca2b expression in rat duodenum. a–d Insm1 expression is scarce (a, b) and mainly localized in duodenal crypts (b, c). Almost no expression can be seen in villous epithelial cells (d). The expression of both Chga (e) and Guca2b (f) is stronger and more widespread compared with that for Insm1. Additionally, Guca2b is mainly expressed in cells of duodenal villi. Bars 50 μm (c, d), 100 μm (b), 200 μm (a, e, f) (GIF 660 kb) [file 441_2016_2393_Fig9_ESM.gif]

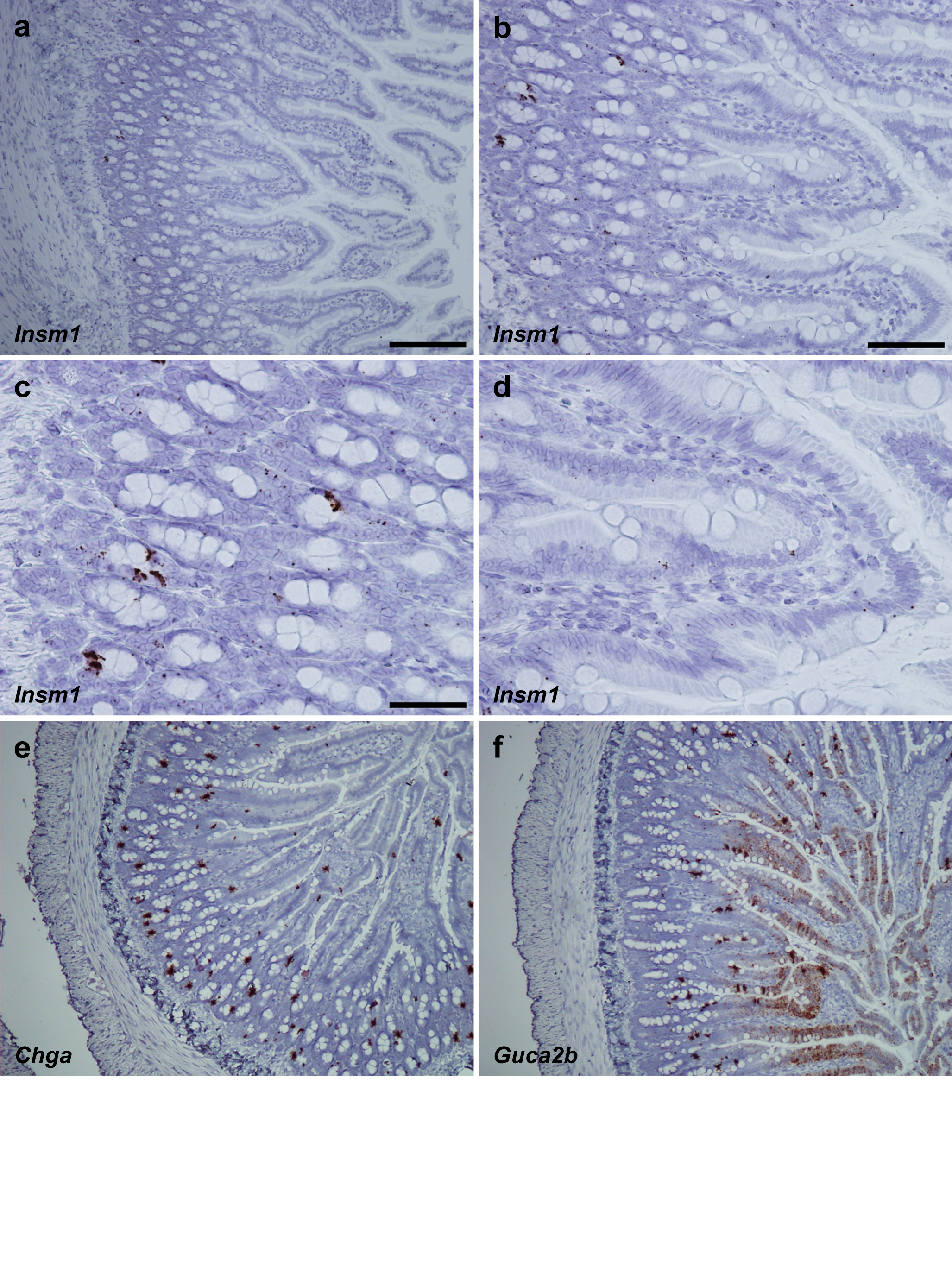

Supplement: Supplementary file 12 — High resolution image file (TIF 8.06 mb) [file 441_2016_2393_MOESM6_ESM.tif]

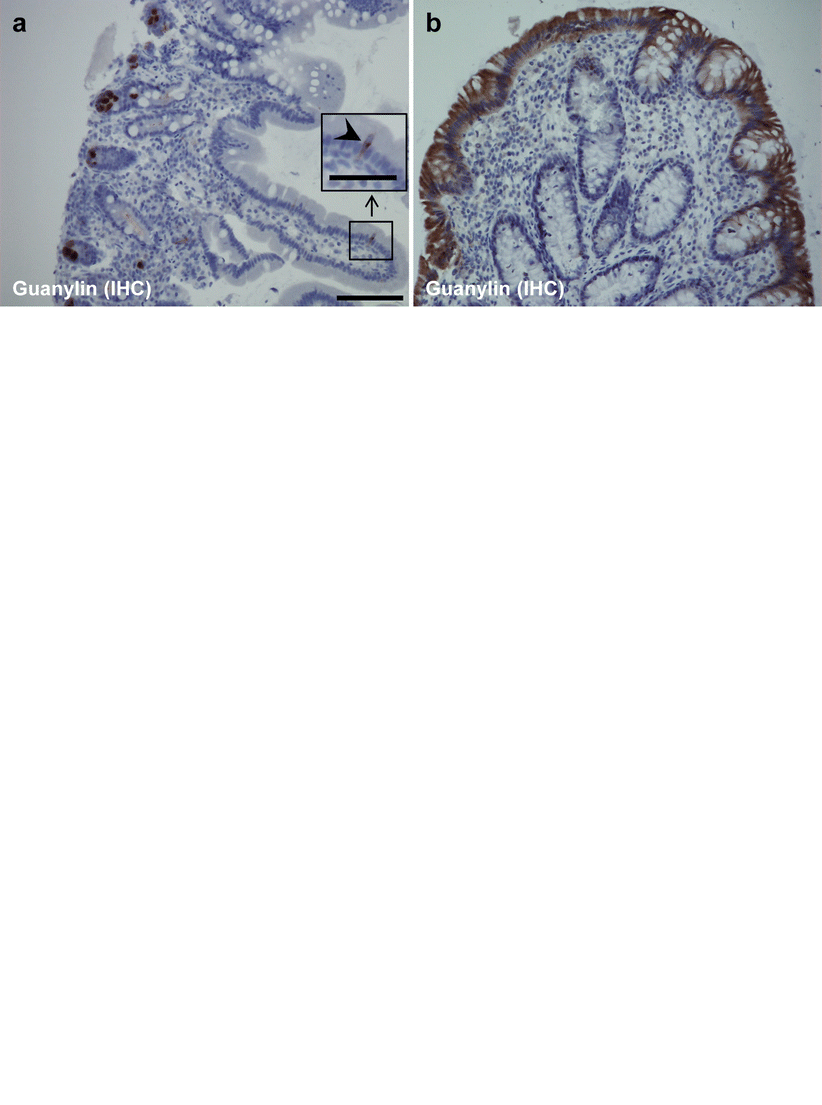

Supplement: Supplementary file 13 — GN (IHC) in human duodenum and colon. a In the duodenum, GN is localized to crypts and occasional epithelial cells of the duodenal villi (insert, arrowhead). b In the colon, strong GN immunoreactivity is seen in the entire superficial epithelial lining. The results of GN IHC are identical to those of GUCA2A ISH. Bars 100 μm (a, b), 50 μm (insert in a) (GIF 225 kb) [file 441_2016_2393_Fig10_ESM.gif]

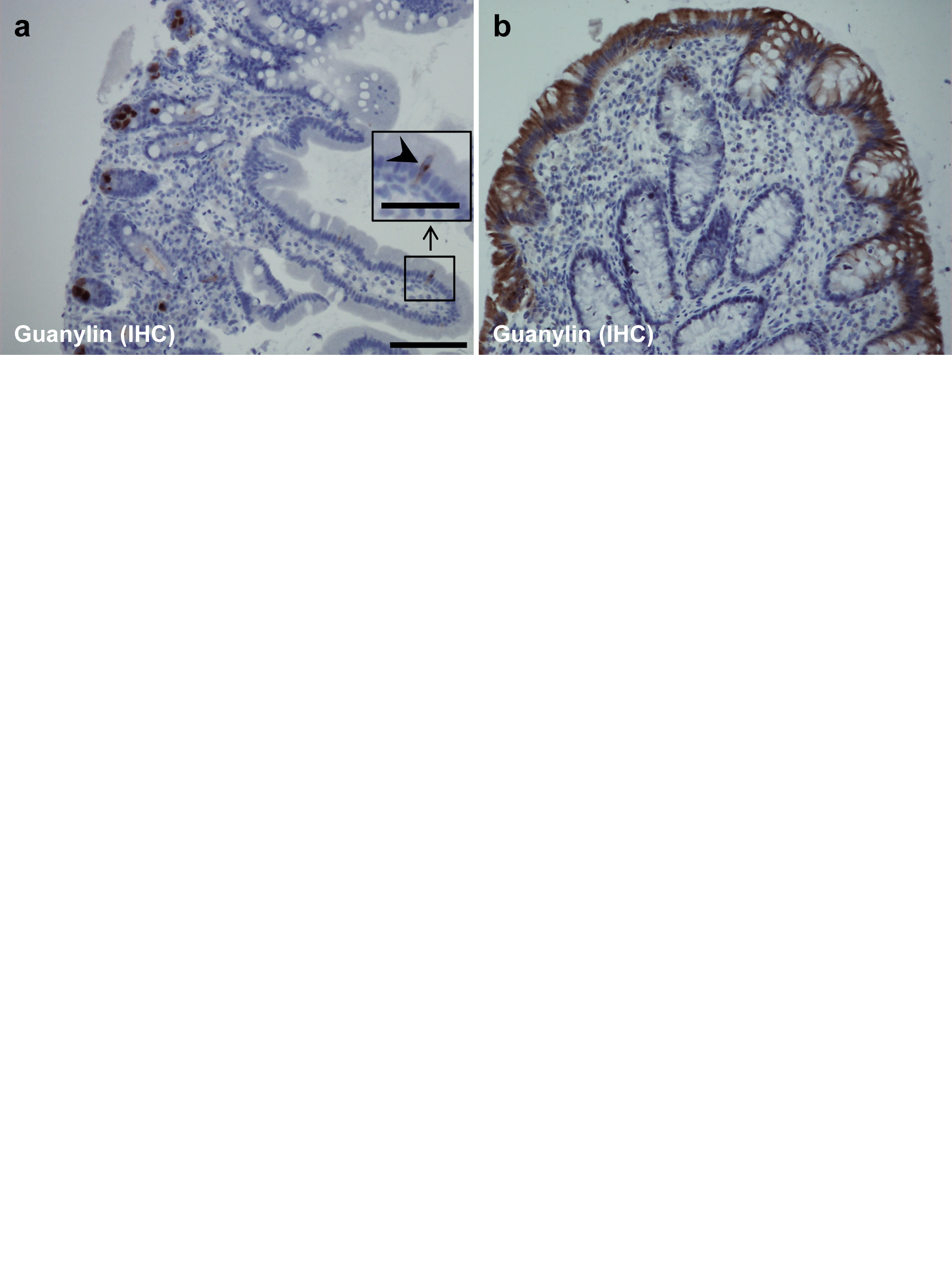

Supplement: Supplementary file 14 — High resolution image file (TIF 2.43 mb) [file 441_2016_2393_MOESM7_ESM.tif]
